# One-year experience of hybrid closed-loop system in children and adolescents with type 1 diabetes previously treated with multiple daily injections: drivers to successful outcomes

**DOI:** 10.1007/s00592-020-01607-4

**Published:** 2020-10-12

**Authors:** Goran Petrovski, Fawziya Al Khalaf, Judith Campbell, Fareeda Umer, Douha Almajaly, Manar Hamdan, Khalid Hussain

**Affiliations:** Division of Endocrinology and Diabetes, Department of Pediatric Medicine, Sidra Medicine, HB 6E 219, Al Luqta Street, Education City North Campus, PO Box 26999, Doha, Qatar

**Keywords:** Type 1 diabetes, Continuous subcutaneous insulin infusion, Continuous glucose monitoring, Diabetes education, Closed-loop systems

## Abstract

**Objective:**

To evaluate the effect of a 1-year hybrid closed-loop (HCL) system on glycemic control in children and adolescents with type 1 diabetes (T1D) previously treated with multiple daily injections (MDI).

**Methods:**

This was a 1-year observational study, as a continuation of the previous 3 months prospective study of pediatric patients with T1D conducted at Sidra Medicine in Qatar. The study enrolled individuals aged 7–18 years with T1D > 1 year, on MDI with self-monitoring of blood glucose or continuous glucose monitoring, with no prior pump experience, and with an HbA1c level < 12.5% (< 113 mmol/mol). After the first 3 months of HCL use, patients were followed at 6, 9 and 12 months, where HbA1c was obtained and pump data were collected.

**Results:**

All 30 participants (age 10.24 ± 2.6 years) who initiated HCL completed 12 months of HCL system use in Auto Mode. The participants used the sensor 88.4 ± 6.5% of the time with Auto Mode usage 85.6 ± 7.4% during 12 months of HCL system use. HbA1c decreased from 8.2 ± 1.4% (66 ± 15.3 mmol/mol) at baseline, to 6.7 ± 0.5% (50 ± 5.5 mmol/mol) at 3 months (*p* = 0.02) and remained stable to 7.1 ± 0.6 (54 ± 6.6 mmol/mol) at 12 months (*p* = 0.02). TIR (70–180 mg/dL) increased from 46.9% at baseline to 71.9% at 1 month and remained above 70% during the 12 months of HCL use.

**Conclusion:**

HCL system (MiniMed 670G) in children and adolescents previously treated with MDI significantly improves glycemic outcomes (HbA1c and Time in Ranges) immediately during the first month. This improved glycemic control was maintained over the 1 year following Auto Mode initiation.

## Introduction

The use of diabetes technology is a promising treatment strategy to improve diabetes outcomes. The use of Continious Subcutaneous Insulin Infusion (CSII) and Continious Glucose Monitoring (CGM) is associated with a reduction in HbA1c, both when used separately and together [1]. Recent technological advances have integrated CSII with CGM, where insulin delivery can be automated by sensor glucose (SG) driven algorithms. Insulin delivery suspension when reaching a low glucose level [2, 3] or in the prediction of a low glucose level [4, 5] has demonstrated a significant reduction in hypoglycemia exposure. Closed-loop systems are designed to maintain glucose levels at a predetermined target by linking CGM information with an insulin dosing algorithm for automated subcutaneous insulin delivery by a pump [6].

The MiniMed 670G [7] is the first hybrid closed-loop (HCL) insulin delivery system for use in diabetes care since 2017 and uses an algorithm to adjust basal insulin delivery automatically every 5 min based on SG values [8]. Several short-term studies on the HLC system have shown improved HbA1c, time in target range, and SG variability in children [9], adolescents and adults [10, 11]. Glycemic outcomes in the pediatric age have been traditionally lower, while the causes are not clear and the underlying causes are to be defined. Three-month observational study [12] has shown that children and adolescents with T1D on MDI therapy without prior pump experience can successfully initiate the HCL system, using a concise structured 10-day protocol and achieve better glycemic outcome than previous treatment with MDI. Another study on the HCL system in children and youth with T1D shows that glycemic control is improved with Auto Mode use when measured by percent TIR, SG and HbA1c during the 6 months follow-up [13].

It remains to be determined whether these short-term effects on improved glycemic control with HCL systems are maintained in the long-term period. Long-term effect (6 and 12 months) of HCL shows scarce results in terms of Auto Mode usage, sensor wear, HbA1c, time in the target range and pump discontinuation [13–16]. The objective of this study was to evaluate the effect of a 1-year HCL system on glycemic control in children and adolescents with T1D previously treated with MDI.

## Methods

This was a 1-year observational study, as a continuation of the previous 3-month prospective study [12] of pediatric patients with T1D conducted at Sidra Medicine in Doha, Qatar. The study enrolled individuals aged 7–18 years with T1D > 1 year, on MDI with SMBG, with or without real-time or intermittent CGM, with no prior pump experience, and with an HbA1c level < 12.5%.

The patient selection, the educational process, as well as the 10-day initiation protocol for the MiniMed 670G HCL system were previously reported [12]. In short, the 10-day initiation protocol consisted of four main stages: Step 1- HCL system compatibility assessment**:** two 1 h introduction sessions in groups of 8–12 patients, where the MiniMed 670G system was described and individuals’ responsibilities and expectations were discussed; Step 2- HCL system training**:** five sessions of 2 h on five consecutive days in groups of 3–5 patients, where CGM was initiated the first day of the training for education and observational purposes and the pump was used as a monitor (insulin delivery with injections); Step 3- Manual Mode**:** HCL system initiation in Manual Mode for 72 h; Step 4- Auto Mode: HCL system initiation and used continuously for 3 months.

After the first 3 months of HCL use, patients would then be seen for routine follow-up at 6, 9 and 12 months.

Demographics and other data (age, gender, and weight, and height, number of visits, diabetes duration and prior CGM use) were collected from the electronic medical record (Power Chart, Cerner Corporation, Kansas City, USA).

Insulin dosing information and CGM data were collected from CareLink Therapy Management Software (Medtronic, Northridge, USA) during the study.

HbA1c was obtained using point of care DCA Vantage Analyzer (Siemens, Erlangen, Germany) at baseline, 3, 6, 9 and 12 months during the study.

The study was approved by the local and National Ethics Committee in Qatar, and all participants and their guardians signed informed consent documents.

### Statistical analysis

Analysis was performed for the entire study population. The data are presented as mean ± SD, median, interquartile or as a percentage. The paired student *t*-test or paired Wilcoxon test, in case of non-normality, was used in the study. A value of 0.05 was considered statistically significant. Statistical analyses were performed using Statistica 12 (Stat Soft, Tulsa, USA).

## Results

### Participants

The 10-day initiation protocol was implemented in 30 participants (age 10.2 ± 2.6 years), and they all completed 12 months of HCL system use in Auto Mode. Baseline characteristics are shown in Table 1.

**Table 1:** Study participant’s characteristics at baseline

	Participants *N* = 30
Age, years	10.2 ± 2.6
Male, *n* (%)	15 (50%)
Female, *n* (%)	15 (50%)
Weight, kg	38.2 ± 12.5
BMI, kg/m^2^	18.6 ± 3.4
BMI, *z*-score	0.2 ± 0.9
Duration of diabetes, years	2.8 ± 1.7
TDD, U/(kg/d)	0.8 ± 0.3
HbA1c, %	8.2 ± 1.4
HbA1c, mmol/mol	66 ± 15.3
Number of visits per patients (3–12 months), *n*	4.8 ± 0,4
Sensor use, *n* (%)	
RT CGM	6 (20%)
isCGM	10 (33%)
No sensor	14 (47%)

All values are shown as mean ± SD, except for gender and sensor use

*BMI* body mass index; *TDD* total daily dose of insulin; *SD* standard deviation; *RT CGM*, real-time continuous glucose monitoring (dexcom G5/guardian connect); is*CGM* intermittent continuous glucose monitoring (freestyle libre)

### Glycemic control

HbA1c decreased from 8.2 ± 1.4% (66 ± 15.3 mmol/mol) at baseline, to 7.1 ± 0.6 (54 ± 6.6 mmol/mol) at 12 months (*p* = 0.02), (Table 2).

**Table 2:** Glucose control, HbA1c, insulin delivered during baseline and study phase

	MDI, Baseline	HCL, 1–3 months	*p*	HCL, 4–6 months	*p*	HCL, 7–9 months	*p*	HCL, 10–12 months	*p*
HbA1c, %	8.2 ± 1.4	6.7 ± 0.8	0.02	6.9 ± 0.7	0.03	6.8 ± 0.5^a^	0.03	7.1 ± 0.6^a^	0.02
HbA1c, mmol/mol	66 ± 15.3	50 ± 8.7		52 ± 7.7		51 ± 5.5		54 ± 6.6	
SG (12 am–12 am), mg/dl	193 ± 41	142 ± 12	0.01	146 ± 20	0.01	141 ± 14	0.01	149 ± 18	0.01
SG (10 pm–06 am), mg/dl	183 ± 35	133 ± 17	0.02	138 ± 16	0.02	132 ± 15	0.01	136 ± 14	0.02
SG (06 am–10 pm), mg/dl	202 ± 42	150 ± 10	0.01	145 ± 17	0.01	149 ± 12	0.02	155 ± 21	0.02
Weight, kg	38.2 ± 12.5	39.4 ± 8.9	0.63	40.2 ± 8.1	0.61	41.7 ± 7.2	0.58	42.8 ± 8.2	0.50
TDD, U/(kg/d)	0.8 ± 0.3	0.9 ± 0.2	0.02	0.9 ± 0.3	0.02	0.9 ± 0.3	0.02	0.9 ± 0.5	0.02
Basal insulin, (12 am–12 am), %	36.5 ± 7.2	42.2 ± 6.7	0.03	43.3 ± 8.8	0.04	42.5 ± 10.2	0.04	43.8 ± 9.8	0.04

Results presented as mean ± SD; *p*-value at 3 months represents change from baseline to 3 months; *p*-value at 6 months represents change from baseline to 6 months; *p*-value at 9 months represents change from baseline to 9 months; *p*-value at 12 months represents change from baseline to 12 months

*SG* sensor glucose; *TDD* total daily dose; *U* units; *d*, day

^a^Due to Covid-19 Pandemic and disruption in clinical services, HbA1c was obtained in 26/30 participants at 9 months and 27/30 participants at 12 months

The aggregated HbA1c over the 12-month period (calculated as the average of HbA1c at 3, 6, 9 and 12 months) was 6.9 ± 0.7 (52 ± 7.7 mmol/mol), significantly lower than 8.2 ± 1.4% (50 ± 5.5 mmol/mol) at baseline (*p* = 0.02).

Mean SG (24 h period) decreased from 193 ± 41 mg/dL at baseline to 149 ± 18 mg/dL during the 12-month period and was similar during each 3-month period (Table 2).

### Time in ranges evolution over time

Time in range (71–180 mg/dL) increased from 46.9% at baseline to 73.4% (*p* = 0.01), over the 12-month period, while time above range (≥ 181 mg/dL) decreased from 49.9% to 23.9% (*p* = 0.01) and time below range did not change significantly.

**Fig 1:**
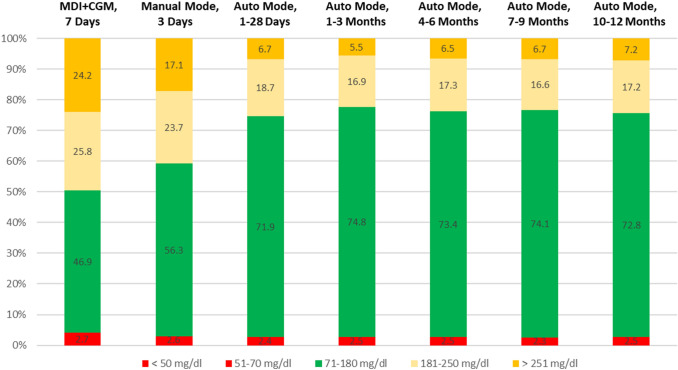
Time in ranges at baseline, during manual mode and auto mode periods. MDI, Multiple Daily Injection; CGM, Continuous Glucose Monitoring results presented as median TIR < 50 mg/dl: MDI + CGM: 0.4%; All other periods 0.3%

Comparing different study periods, TIR (70–180 mg/dL) increased from MDI + CGM to Manual Mode (*p* = 0.01); Manual Mode to Auto Mode of 28 days (*p* = 0.01); Auto Mode of 28 days to 3 months (*p* = 0.72); Auto Mode of 3 months to 6 months (*p* = 0.67); Auto Mode of 6 months to 9 months (*p* = 0.81) and Auto Mode of 9 months to 12 months (*p* = 0.77), as shown in Fig. 1.

Time above range (≥ 181 mg/dL), decreased from 50% on MDI + CGM to 25.4% at Auto Mode of 28 days (*p* = 0.02), and was maintained below 25% during the study.

### Therapy-related changes

TDD (Total Daily Dose) delivered, increased by 0.1 U/kg (*p* = 0.02) during the study, compared to baseline (Table 2) and the percent of basal insulin out of the TDD increased significantly from 36.5 ± 7.2 to 43.8 ± 9.8% during this interval.

Insulin to Carbohydrate Ratio (ICR) during the initial Auto Mode period was made more aggressive regardless of the meal period compared with baseline and decreased from 16.2 ± 5.7 g at the beginning to 12.8 ± 4.8 g at the third month of Auto Mode (*p* = 0.01), to 10.1 ± 6.2 g at the end of the study (*p* = 0.01). ICR in participants with Hba1c > 8.5% (69 mmol/mol) at baseline was significantly higher, 17.8 ± 6.4 g compared to 14.6 ± 5.2 g in participants with HbA1c < 7.0% (53 mmol/mol) at baseline (*p* = 0.04), without significant difference 13.2 ± 5.6 g to 12.5 ± 4.2 at third month (*p* = 0.58), and 9.8 ± 6.4 g to 10.3 ± 6.1 g at the end of the study (*p* = 0.76), respectively.

### HCL system usability

The participants used the sensor of 89.8 ± 7.5% of the time in the first 2 weeks after activating Auto Mode and did not change over the 12-month period, while Auto Mode usage remained constant above 85%.

The total number of Auto Mode exits significantly decreased between the first 2 weeks and the first 3 months after enabling Auto Mode, from 8.4 ± 1.8 to 4.2 ± 0.9 events per week, respectively (*p* = 0.01), and remained stable during other study periods (Table 3).

**Table 3:** HCL system characteristics during the auto mode period

	HCL, 2 weeks	HCL, 3 months	*p*	HCL, 6 months	*p*	HCL, 9 months	*p*	HCL, 12 months	*p*
Sensor wear, %	89.8 ± 7.5	92.4 ± 6.2	0.49	90.8 ± 4.9	0.42	89.7 ± 8.2	0.55	88.4 ± 6.5	0.44
Auto Mode, %	84.8 ± 11.9	89.8 ± 7.4	0.78	89.6 ± 5.8		88.9 (9.1)	0.67	85.6 ± 7.4	0.06
Calibration, *n* per day	4.3 ± 1.2	3.4 ± 0.9	0.01	3.5 ± 0.8		3.1 ± 0.9	0.02	3.3 ± 1.1	0.03
Carbohydrates, g per day	213 ± 50	219 ± 48	0.70	232 ± 37	0.72	244 ± 58	0.71	256 ± 55	0.82
Carb ratio, g	16.2 ± 5.7	12.8 ± 4.8	0.01	11.9 ± 5.1	0.02	10.8 ± 4.2	0.02	10.1 ± 6.2	0.01
Active insulin time, h	3.6 ± 0.4	3.5 ± 0.4	0.15	3.5 ± 0.5	0.18	3.5 ± 0.9	0.16	3.4 ± 1.1	0.21
Auto mode exits per patient/week	8.4 ± 1.8	4.2 ± 0.9	0.01	3.8 ± 1.2	0.01	4.4 ± 1.4	0.02	4.7 ± 2.0	0.02

Results presented as mean ± SD; *p*-value at 3 months represents change from baseline to 3 months; *p*-value at 6 months represents change from baseline to 6 months; *p*-value at 9 months represents change from baseline to 9 months; *p*-value at 12 months represents change from baseline to 12 months

The number of daily calibrations also significantly decreased from 4.3 ± 1.2 in the first 2 weeks to 3.4 ± 0.9 at the first 3 months of Auto Mode use (*p* = 0.01) and remained similar throughout the 12 months. The number of meals and carbohydrate intake did not differ from the beginning to the end of the study.

### Safety

There was no event of severe hypoglycemia, diabetic ketoacidosis (DKA) or hospital admission during the study. There were six episodes of hyperglycemic events (infusion blockage 4 and influenza 2) that needed medical attention by phone consultation with the diabetes team.

## Discussion

This is the first 1-year study evaluating the effect of the HCL system on glycemic control in children and adolescents with T1D transitioning from MDI to advanced insulin delivery systems. The excellent outcomes and high retention of the participants for what is conceived as a complex therapy can serve as a model for practices treating adolescents with T1D.

As the sensor wear time drives, the Auto Mode usage is required for better outcomes, and the 88.4 ± 6.5% sensor usage achieved at 12 months was one of the important achievements. This high percentage use of automation and no dropout rates can be attributed to the specific selection/initiation protocol including the initial use of the system as a sensor alone for 7 days, although the relative contribution of each component cannot be determined from the study. The study confirmed previous conclusions, that increased Auto Mode usage is associated with lower HbA1c and increased TIR, resulting in that patients with both higher sensor wear times and increased Auto Mode usage had incrementally lower HbA1c than patients with lower sensor wear time and less Auto Mode usage [17]. Our data showed high Auto Mode usage of 85.6% at 1 year, which is higher than 51% at 6 months after HCL initiation [13].

All 30 individuals enrolled in the 3-month prospective study [12] completed 1-year usage of the MiniMed 670G system with Auto Mode, and no attrition was observed, compared to 46% of participants, who stopped using the Auto Mode in the 1-year period reported by Lal et al. [16] and 19% discontinuation rate after 8 months, reported by Godwin et al. [18]. This can be partly due to the low number of Auto Mode exits, averaged 4.7 ± 2.0 per patient per week during the 12-month period, similar to 4.8 ± 1.6 exits at 3 months [9], which indicates that the participants were educated appropriately, and the system settings were optimized to reduce exits thus the system use became more comfortable over time. Participants’ high engagement and no attrition attest the effectiveness of the structured initiation HCL protocol, where realistic expectations and patient responsibilities were discussed and evaluated, as well as reflecting indirectly on the patient's and caregiver’s satisfaction with the clinical and patients related outcomes.

This observational study confirms the potential of HCL systems to improve overall glycemic control [19, 20]. The 12 months observational results provide evidence for the sustainability of these results beyond the initial 3-month period reported previously [12] and are superior to previously reported outcomes in children [9] and adolescents [11]. The improved HbA1c in our study after 3 months remained stable in the long term with 7.1 ± 0.6% (54 ± 6.6 mmol/mol) at 1 year, compared to 7.6 ± 0.8 (60 ± 8.7 mmol/mol) at 1 year [16] and 7.2 ± 0.1 (55 ± 1.1 mmol/mol) at 6 months of HCL initiation [14] in previous reports. The HbA1c target of < 7.0% (53 mmol/mol) established by the ADA and ISPAD guidelines for glycemic control in children [21, 22] was reached in 73% of the participants at the end of the study.

The International Consensus on CGM recommends that CGM metrics such as TIRs should be complementary to HbA1c in patient care [23]. The time spent within, below and above the target glycemic range have been defined as glycemic goals, beyond HbA1c [24, 25]. In our study, TIR (70–180 mg/dL) significantly increased to 74.8% at 3 months of Auto Mode use, which is higher than 68.8% in adolescents [11] and 64.6% in children [9] for the same study period. TIR (70–180 mg/dL) remained above 70% during the 1-year period, which is similar to 70.1% in adults [14], but significantly higher than 56.9% in youth [13], both at 6 months of HCL initiation. Time in range, time below range and time above range observed in our study, all achieved the desired clinical targets for CGM data interpretation [26].

The superior clinical outcomes in our study were most probably driven by the high sensor and Auto Mode use, and can be attributed to our specific selection/initiation protocol, the parental/guardian involvement and supervision of the children, as well as the support and follow-up by the diabetes team. The 10-day initiation HCL protocol for individuals on MDI enables the activation of the Auto Mode feature after 3 days of Manual Mode use, which is significantly shorter compared to 2–4 weeks, reported in other studies [9, 11, 27]. This shortened process might be better accepted by those initiating the technology. Proper settings of the pump in order to decrease patient being burdened by exits and additional SMBG requirements are important elements in the initiation and follow-up process. Modifying the ICR by increasing the meal bolus dose by almost 20% during the first month of Auto Mode use is necessary when initiating individuals on the HCL system from MDI regimens, which is similar to previous reports [28]. Our study shows that further strengthening of ICR was needed on the long-term period, as the TDD of insulin increases in children due to their growth and evaluation on ICR should be performed on each visit.

The observational period provided additional insight into the drivers for the improved clinical outcomes bestowed by the automated system. Both TDD and the percentage of daily basal insulin delivered, slightly increased, and the percentage of nightly basal insulin delivered slightly decreased, which effectively distributed the insulin delivery according to patients’ individual requirements, resulting in better control with minimal increase in total insulin dosages.

The improved clinical outcomes observed in our study were achieved in a safe manner, with no events of DKA, or severe hypoglycemia, and with no hospital admission. Several hyperglycemic events, which were noted during the study can be considered as regular issues in diabetes management in the real world. These events were managed by phone consultations without any impact on glycemic control or quality of life in the participants.

We acknowledge several limitations of our study: single center, absence of a control group and relatively small number of participants. We did not analyze the quality of life, patient-reported outcomes and psycho-social and economic factors of the HCL system use. However, the objective of this study was to evaluate the long-term (1 year) effect of the HCL system on glycemic control.

## Conclusion

In conclusion, whereas HCL systems, like the MiniMed 670G system, have demonstrated the potential to improve glycemic outcomes in the short-term period, our findings confirm that these improvements are maintained over a 1-year period following Auto Mode initiation. All individuals enrolled in this study completed 1-year usage of the MiniMed 670G system with Auto Mode with no dropouts, unlike the previously reported studies and a significant decrease of the calibration was observed. In our clinical practice, patients’ selection criteria, concise initiation protocol, participants’ high engagement with the HCL system as well as structured follow-up, and appropriate pump settings were key contributors to achieve and maintain better glycemic control. Further investigation on a more varied larger population and patient satisfaction should be performed to confirm these findings.
